# Scene in an Open-Air Pharmacy

**DOI:** 10.4103/1995-705X.73226

**Published:** 2010

**Authors:** Rachel Hajar

**Figure:**
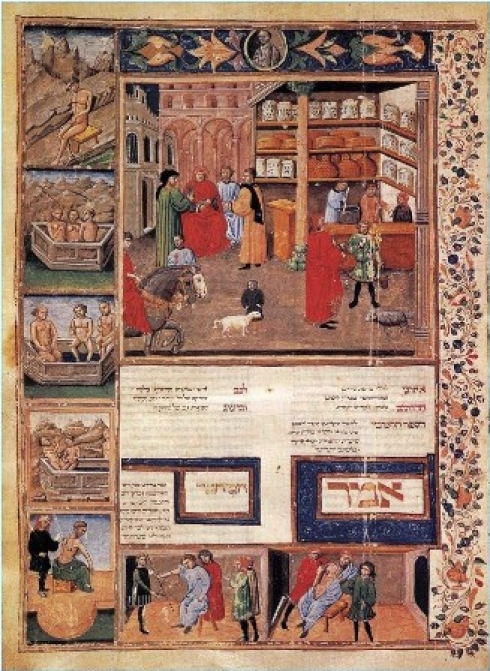
A pharmacy and many scenes of routine healthcare illuminated manuscript illustration from Canon of Medicine by Avicenna, c.1440, Bibiloteca Universitaria, Bologna, Italy

